# Metallomic and Untargeted Metabolomic Signatures of Human Milk from SARS‐CoV‐2 Positive Mothers

**DOI:** 10.1002/mnfr.202200071

**Published:** 2022-06-29

**Authors:** Ana Arias‐Borrego, Francisco J. Soto Cruz, Marta Selma‐Royo, Christine Bäuerl, Elia García Verdevio, Francisco J. Pérez‐Cano, Carles Lerin, Inés Velasco López, Cecilia Martínez‐Costa, M. Carmen Collado, Tamara García‐Barrera

**Affiliations:** ^1^ Research Center for Natural Resources, Health and the Environment (RENSMA) Department of Chemistry Faculty of Experimental Sciences. Fuerzas Armadas Ave University of Huelva Huelva 21007 Spain; ^2^ Department of Analytical Chemistry, Faculty of Chemistry University of Seville Professor García González Ave. Seville 41012 Spain; ^3^ Institute of Agrochemistry and Food Technology‐National Research Council (IATA‐CSIC) Agustin Escardino 7, 46980 Paterna Valencia Spain; ^4^ Department of Gynecology and Obstetrics Hospital Universitario Doctor Peset Valencia 46017 Spain; ^5^ Physiology Section Department of Biochemistry and Physiology Faculty of Pharmacy and Food Science University of Barcelona (UB) Barcelona 08028 Spain; ^6^ Nutrition and Food Safety Research Institute (INSA‐UB) Santa Coloma de Gramenet 08921 Spain; ^7^ Endocrinology Department Institut de Recerca Sant Joan de Déu Hospital Sant Joan de Déu Barcelona 08950 Spain; ^8^ Department of Gynecology & Obstetrics Hospital Universitari Germans Trias i Pujol s/n Carretera del Canyet Badalona 08916 Spain; ^9^ Department of Pediatrics University of Valencia. INCLIVA Biomedical Research Institute Avenida Blasco Ibáñez 15‐17 Valencia 46010 Spain

**Keywords:** COVID‐19, elements, human milk, metabolomics

## Abstract

**Scope:**

Lack of information about the impact of maternal severe acute respiratory syndrome coronavirus 2 (SARS‐CoV‐2) infection on the elemental and metabolomic profile of human milk (HM).

**Methods and results:**

An observational study on HM from mothers with COVID‐19 is conducted including a prepandemic control group. Maternal–infant clinical records and symptomatology are recorded. The absolute quantification of elements and untargeted relative metabolomic profiles are determined by inductively coupled plasma mass spectrometry and gas chromatography coupled to mass spectrometry, respectively. Associations of HM SARS‐CoV‐2 antibodies with elemental and metabolomic profiles are studied. COVID‐19 has a significant impact on HM composition. COVID‐19 reduces the concentrations of Fe, Cu, Se, Ni, V, and Aluminium (Al) and increases Zn compared to prepandemic control samples. A total of 18 individual metabolites including amino acids, peptides, fatty acids and conjugates, purines and derivatives, alcohols, and polyols are significantly different in HM from SARS‐CoV‐2 positive mothers. Aminoacyl‐tRNA biosynthesis, phenylalanine, tyrosine and tryptophan biosynthesis, phenylalanine, and linoleic acid pathways are significantly altered. Differences are obtained depending on COVID‐19 symptomatic and asymptomatic status.

**Conclusions:**

This study provides unique insights about the impact of maternal SARS‐CoV‐2 infection on the elemental and metabolomic profiles of HM that warrants further research due the potential implications for infant health.

## Graphical abstract

Maternal COVID‐19 infection reduces the human milk levels of Fe, Cu, Se, Ni, V, and Al, and increases Zn, compared to prepandemic control samples. The untargeted metabolomic profile reveals alterations in the aminoacyl‐tRNA, tyrosine and tryptophan, phenylalanine, and linoleic acid pathways. The study also reports differences depending on symptomatic and asymptomatic status and between different lactational stages as well as associations of human milk elements with metabolites and antibodies.

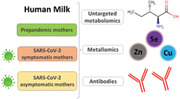

## 1 Introduction

Breastfeeding is considered the gold standard for infant feeding and is of crucial importance in influencing both, infant growth and development, as well as prevention of future diseases during adulthood. The COVID‐19 pandemic has led to concerns over mother‐to‐child transmission, and breastfeeding practices have being drastically reduced^[^ ^1^ ^]^; with potential consequences on child health, especially regarding its protection against infections. Current evidence shows that human milk (HM) is not a transmission vehicle for severe acute respiratory syndrome coronavirus 2 (SARS‐CoV‐2)^[^ ^2^, ^3^ ^]^ however, the maternal exposure to the virus induce an antibodies (Ab) response against it.^[^ ^4^ ^]^

HM contains, beyond nutritional aspects, a complex combination of nutrients (macro‐ and micronutrients) and also, bioactive components, including immunoglobulins, oligosaccharides, microorganisms, and metabolites among others.^[^ ^5^, ^6^ ^]^

The relevance of elements is essential as about one‐third of human proteins need their presence to develop their function.^[^ ^7^ ^]^ Serum levels of Cu, Zn, and other biometals have been associated with COVID‐19 severity.^[^ ^8^ ^]^ Higher serum Se levels have been found in surviving COVID‐19 patients compared to those reported in nonsurviving patients.^[^ ^9^, ^10^, ^11^ ^]^ Limited evidence has been reported on elements transfer during breastfeeding^[^ ^12^, ^13^, ^14^, ^15^, ^16^ ^]^ and the impact of maternal SARS‐CoV‐2 infection on HM elemental and metabolomic profiles are uncovered.

Thus, we analyzed the HM elements profile from SARS‐CoV‐2 positive mothers by inductively coupled plasma mass spectrometry (ICP‐MS). A metabolomic study was also carried out to delve into the metabolome of HM from SARS‐CoV‐2 positive mothers as well as the possible link between metallomic and metabolomic profiles of HM. This study goes deeper into the impact of maternal SARS‐CoV‐2 infection on the HM elemental and metabolomic profile and compares to the prepandemic control milk samples. In addition, the presence of symptoms during SARS‐CoV‐2 infection and the potential metabolic alterations were assessed.

## 2 Results

### 2.1 Study Population Characteristics

No significant differences in clinical variables were observed between groups with the exception of the age (*p* = 0.002) (**Table** **1**). Among the COVID‐19 women, 28 were diagnosed with specific SARS‐CoV‐2 PCR test on nasopharyngeal swabs while six were seropositive (IgG‐positive). PCR tests were performed as part of routine surveillance before delivery at hospital. Furthermore, 16 (47.1%) COVID‐19 women were asymptomatic, and the rest reported mild COVID‐19 symptoms (pain, fatigue, or headache, among others). No other effects or medical problems were reported. All neonates were negative for SARS‐CoV‐2 and in good health.

**Table 1:** Characteristics of the volunteers included in the study

	COVID‐19 (*n* = 35)	Prepandemic control (*n* = 20)	*p*‐value
Maternal characteristics:			
Age	35.2 ± 4.4	30.8 ± 5.3	0.002
Gestational age [weeks]*	39.2 ± 1.7	39.3 ± 1.1	0.833
Vaginal birth, n [%]	28/35 (80%)	14/20 (70%)	0.522
Infant characteristics:			
Birth weight [g]	3292 ± 577	3374 ± 406	0.578
Birth length [cm]	49.5 ± 2.6	50.9 ± 1.7	0.158
Breastfeeding status			
Exclusive	27 (77.14 %)	16 (80.0%)	<0.999
Mixed feeding	8 (22.86 %)	4 (20.0%)	
Gender, male [%]	17/35 (48.6%)	12/20 (60%)	0.575

^*^Values are given as mean and standard deviation.

Statistical differences between categories in quantitative and qualitative data were assesed by Unpaired t‐test and Fisher's exact test (two‐sided), respectevely.

### 2.2 Human Milk Metallomic Profile is Influenced by SARS‐CoV‐2 Infection

**Figure 1:**
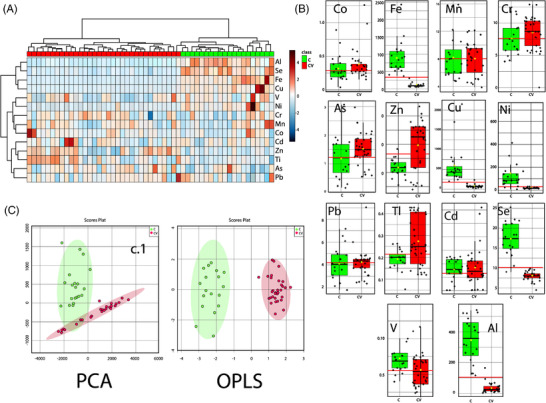
Impact of SARS‐CoV‐2 infection of human milk elemental profile. **A)**. Heatmap showing the differential elemental profile of HM from COVID‐19 mothers against controls (*p* < 0.05) (adjusted by FDR correction). **B)**. Box plots of levels determined for 14 elements of HM from COVID‐19 mothers compared to controls. **C)**. C.1) Principal component analysis (PCA) and C.2) Scatter plot of orthogonal discriminant analysis (OPLS‐DA). Classification based on their elemental profile of HM from C: healthy controls and CV: SARS‐CoV‐2 positive mothers with symptoms and asymptomatic.

HM metallomic profile from SARS‐CoV‐2 positive mothers had significantly different concentrations of nine elements of the 14 analyzed by ICP‐MS compared to prepandemic women. There was a higher inter‐ and intravariability in the metallomic profile (**Figure** **1**A). In general, most of the elements (Se, Ni, Fe, Cu, V, and Al) were found in significant lower concentrations in COVID‐19 mothers compared to controls (Figure 1B, **Table** **2**), except zinc (1.7‐fold, *p* = 0.0001) and also, some minor metals as thallium (1.4‐fold, *p* = 0.008) and arsenic (1.3‐fold, *p* = 0.031). The AUC values highlight the strong association of COVID‐19 with altered HM elemental profile (Table 2).

**Table 2:** Concentration of elements in the analyzed samples in comparison with previously reported levels in human milk from healthy women

Elements	Averaged concentration [ng g^–1^] ± S.E.M.		Range concentration from literature [ng g^–1^ or ng mL^–1^]	Fold change [*p*‐values]	ROC [AUC]
	CV	C		CV/C	CV versus C
Zn	3949.5 ± 0.4	2324.6 ± 0.3	255–6970	**1.7 (*p* = 0.000)**	0.79
Fe	83.4 ± 0.6	883.6 ± 0.5	47–1720	**0.1 (*p* = 0.000)**	1
Cu	66.4 ± 0.5	741.4 ± 1.2	56–760	**0.1 (*p* = 0.000)**	1
Al	35.2 ± 0.8	348.7 ± 0.3	7.06–890	**0.1 (*p* = 0.000)**	0.98
Ni	22.9 ± 1.1	144.8 ± 1.2	0.79–480	**0.2 (*p* = 0.000)**	0.91
Cr	8.5 ± 0.3	7.7 ± 0.3	0.17–24.3	1.1 (*p* = 0.105)	0.64
Mn	8.3 ± 0.3	8.2 ± 0.4	0.929–133	1.0 (*p* = 0.501)	0.56
Se	8.0 ± 0.1	17.4 ± 0.2	5.6–32.1	**0.5 (*p* = 0.000)**	0.99
Pb	4.2 ± 0.3	4.1 ± 0.4	0.019–18.17	1.0 (*p* = 0.470)	0.55
As	1.5 ± 0.3	1.2 ± 0.6	0.016–7.8	**1.3 (*p* = 0.031)**	0.67
V	0.6 ± 0.4	0.7 ± 0.3	0.18–1.2	**0.8 (*p* = 0.011)**	0.71
Cd	0.5 ± 0.6	0.5 ± 0.3	0.003–1.37	1.1 (*p* = 0.501)	0.53
Co	0.4 ± 0.6	0.3 ± 0.6 (97)	0.01–0.85	1.2 (*p* = 0.067)	0.64
Tl	0.3 ± 0.4	0.2 ± 0.2	0.04 ± 0.01	1.4 (*p* = 0.008)	0.72

ROC, AUC: area under the receiver operating characteristic curve; SEM, standard error of the mean. C: Controls (HM from healthy women), CV: HM from SARS‐CoV‐2 positive mothers.

Elements were identified in all samples analyzed with exception of Al (94% in CV), Cr (97% in CV), and Co (97% in C).

The PCA based on the elemental composition allowed distinct clustering of the HM samples from COVID‐19 mothers to the healthy controls (Figure 1C). The first two components (PC1 and PC2) accounted for 95% of the total variance with less contribution of the other components (Figure S2, Supporting Information). The OPLS‐DA model confirmed the clear separation of the HM from COVID‐19 and control group (Figure 1C). In addition, no different clustering was observed when asymptomatic and symptomatic milk samples from COVID‐19 women were compared. Furthermore, considering the lactational stage, we also identified a distinct profile between control and symptomatic SARS‐CoV‐2 mothers (Figure S3, Table S3, Supporting Information).

### 2.3 Untargeted Metabolomic Profile of Human Milk Samples from SARS‐CoV‐2 Positive Mothers

**Figure 2:**
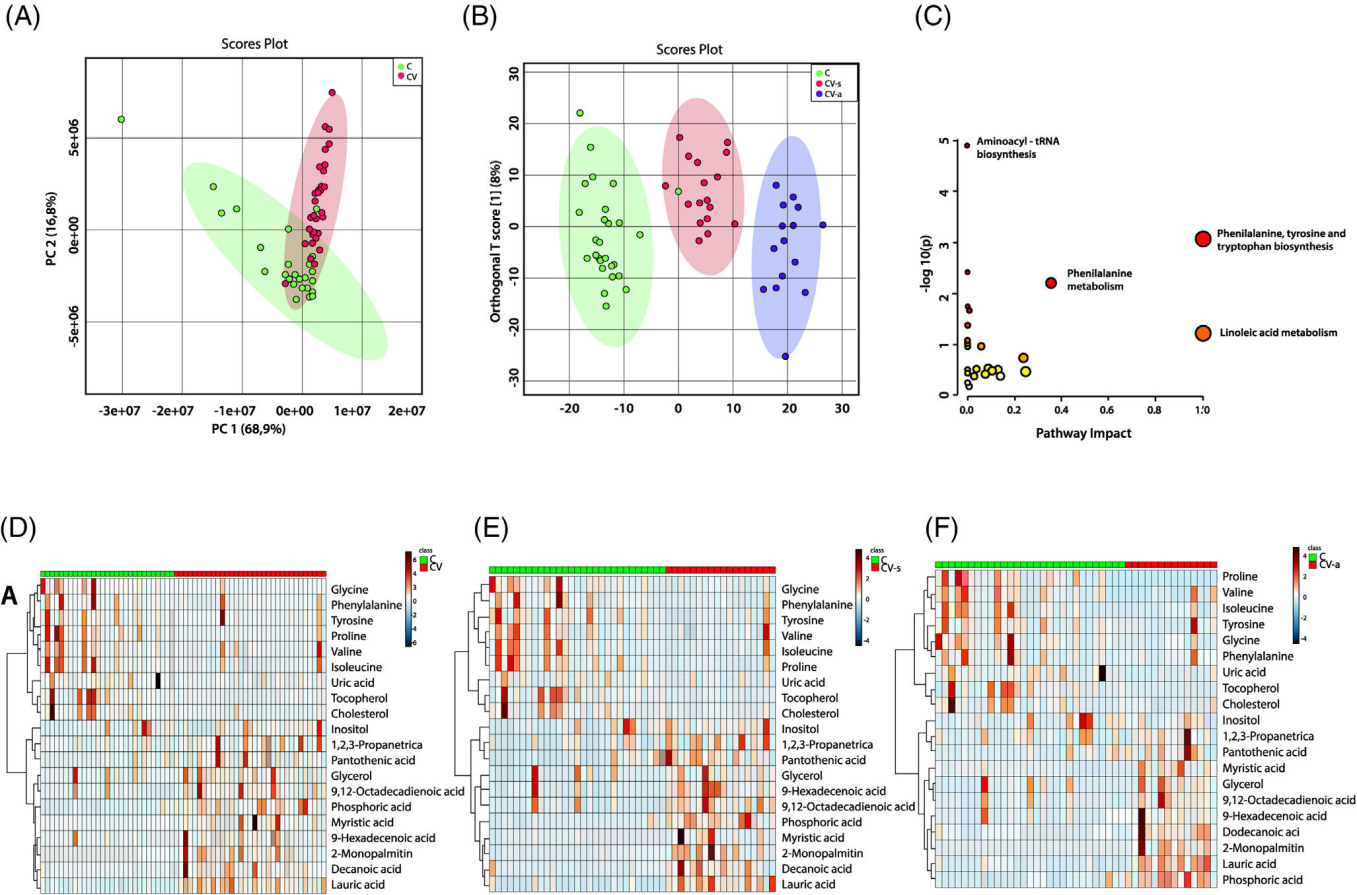
Impact of SARS‐CoV‐2 infection on human milk metabolite profile. **A**) Classification samples based on their metabolomic profile of HM from C: healthy controls, CV‐s: SARS‐CoV‐2 positive mothers with symptoms and CV‐a: SARS‐CoV‐2 positive asymptomatic mothers by PCA‐3D and **B**) orthogonal partial least squares‐discriminate analysis (OPLS‐DA) plot of differential metabolites. **C**) Summary chart of the interconnected metabolic pathways related to altered metabolites in human milk of SARS‐CoV‐2 positive mothers. *p*‐value is the *p* calculated from the enrichment analysis and the impact is the pathway impact value calculated from pathway topology analysis. **D)** Distinct metabolomic profiles according COVID‐19 and control groups. Heatmaps showing the differential metabolomic profile of HMB from COVID‐19 mothers against controls. **E)** COVID‐19 mothers with symptoms against controls and **F)** COVID‐19 mothers asymptomatic against controls (*p* < 0.05). C: HM from healthy controls, CV‐s: HM from SARS‐CoV‐2 positive mothers with symptoms, and CV‐a: HM from SARS‐CoV‐2 positive asymptomatic mothers.

A total of 18 individual metabolic features were significantly different between SARS‐CoV‐2 positive mothers and control (**Table** **3**). Differences between symptomatic and asymptomatic COVID‐19 and controls were identified (Table 3). PCA allowed observing the separation of HM samples from SARS‐CoV‐2 positive mothers and controls based on their metabolomic profile (**Figure** **2**A) and the first two components explained 95.1% of the total variance (PC1 = 94.5%). Score plots of OPLS‐DA obtained by MSGas cromatography‐mass spectrometry(GC‐MS) allowed a perfect classification between HM from SARS‐CoV‐2 positive mothers with symptoms, asymptomatic, and controls (Figure 2B). In addition, the parameters of quality confirmed the discrimination power of the models: R^2^Y = 0.988, *Q* ^2^ = 0.699 for GC−MS. Once that the classification of the samples was confirmed, the *m*/*z* peaks responsible for the grouping of samples were identified. HM distinct metabolites identified as biomarkers of COVID‐19 in lactating women (with symptoms and asymptomatic) are shown in Table 3. The most altered pathways altered in HM due COVID‐19 were aminoacyl‐tRNA biosynthesis, phenylalanine, tyrosine and tryptophan biosynthesis, phenylalanine metabolism, and linoleic acid metabolism (Figure 2C). Furthermore, the differential metabolomic profile of HM from SARS‐CoV‐2 positive mothers with symptoms, asymptomatic, and controls were identified (Figures 2D–F).

**Table 3:** Altered HM metabolites in COVID19 women ordered by class

Compound	Accession numbers	Category	Mass human database	RT [min]	Kovat retention index	Targeted ions (*m*/*z*)	XTMS	CV in QC [%]	*p‐value C versus CV‐s	*p‐value C versus CV‐a	*p‐value C versus CV	Regulation	AUC C versus CV	AUC C versus CV‐a	AUC C versus CV‐s	FC C versus CV‐a	FC C versus CV‐s	FC C versus CV
Valine	HMDB0000883	Amino acids, peptides and analogues	117.079	8.90	1193	144, 149, 218	2TMS	4	0.096	0.141	**0.016**	↓	0.73	0.70	0.74	0.44	0.42	0.43
Glycerol	HMDB0000131	Carbohydrates and carbohydrate conjugates	92.0473	6.62	1241	218, 293	3TMS	3	0.185	**0.048**	0.014	↑	0.81	0.79	0.83	2.83	2.27	2.53
Isoleucine	HMDB0000172	Amino acids, peptides and analogues	131.0946	9.93	1262	158, 160, 218	2TMS	14	**0.029**	**0.038**	**0.003**	↓	0.78	0.77	0.80	0.22	0.23	0.22
Proline	HMDB0000162	Amino acids, peptides and analogues	115.0633	10.05	1270	142, 216, 244	2TMS	10	**0.005**	**0.004**	**0.000**	↓	0.81	0.81	0.80	0.05	0.13	0.10
Glycine	HMDB0000123	Amino acids, peptides and analogues	75.0320	10.13	1275	174, 248, 277	3TMS	20	**0.037**	**0.039**	**0.003**	↓	0.74	0.76	0.73	0.30	0.33	0.31
Decanoic acid	HMDB0000511	Fatty acids and conjugates	172.1463	12.17	1410	117, 129, 229	1TMS	21	**0.014**	**0.000**	**0.000**	↑	0.94	0.98	0.91	7.90	4.69	6.15
Phenylalanine	HMDB0000159	Amino acids, peptides and analogues	165.079	14.22	1855	192, 218, 266, 294	2TMS	12	0.102	0.284	**0.030**	↓	0.72	0.70	0.75	0.40	0.23	0.31
Lauric acid	HMDB0000638	Fatty acids and conjugates	200.1776	14.52	1948	75, 129, 257	1TMS	13	**0.000**	**0.000**	**0.000**	↑	0.96	0.97	0.95	7.17	5.08	6.03
Phosphoric acid	HMDB0001429	Non‐metal phosphates	97.9769	15.60	2114	299, 357, 445	4TMS	7	**0.000**	**0.000**	**0.000**	↑	0.94	0.94	0.94	7.25	8.77	8.08
1,2,3‐Propanetricarboxylic acid	HMDB0031193	Tricarboxylic acids and derivatives	176.1241	16.22	2145	211, 273, 347465	4TMS	21	**0.003**	0.390	**0.005**	↑	0.72	0.69	0.74	1.91	3.25	2.64
Tyrosine	HMDB0000158	Amino acids, peptides and analogues	181.0739	17.53	2207	218, 280, 354	3TMS	15	0.151	0.439	0.059	↓	0.72	0.74	0.70	0.55	0.34	0.44
Pantothenic acid	HMDB0000210	Alcohols and polyols	219.1107	18.03	2243	201, 291, 420	3TMS	5	**0.007**	**0.004**	**0.000**	↑	0.78	0.75	0.81	3.79	3.47	3.62
Inositol	HMDB0006088	Alcohols and polyols	180.0634	18.32	2147	191, 305, 318, 507	6TMS	4	0.222	0.858	0.449	↑	0.61	0.52	0.72	0.75	1.74	1.29
9‐Hexadecenoic acid	HMDB0003229	Fatty acids and conjugates	254.2246	18.43	1712	129, 199, 311	1TMS	11	0.077	**0.012**	**0.003**	↑	0.85	0.82	0.88	4.42	3.40	3.86
Uric acid	HMDB0000289	Purines and purine derivatives	168.0283	19.03	2314	441, 456, 460	4TMS	8	0.171	**0.035**	**0.011**	↓	0.77	0.88	0.68	0.19	0.45	0.33
9,12‐Octadecadienoic acid	HMDB0000673	Lineolic acids and derivatives	280.2402	20.13	2015	178, 262, 337	1TMS	9	**0.008**	**0.001**	**0.000**	↑	0.88	0.87	0.89	4.10	3.43	3.74
Myristic acid	HMDB0000806	Fatty acids and conjugates	228.2089	21.62	2044	203, 211, 343, 341	2TMS	13	**0.005**	0.162	**0.003**	↑	0.80	0.80	0.79	9.48	15.30	12.66
2‐Monopalmitin	HMDB0011533	Monoradylglycerols	330.2770	22.87	2068	218, 313, 459	2TMS	21	**0.020**	**0.000**	**0.000**	↑	0.91	0.91	0.90	21.68	10.98	15.84
Tocopherol	HMDB0002902	Quinone and hydroquinone lipids	402.3498	27.07	3254	237277, 502, 503	1TMS	16	**0.012**	**0.009**	**0.001**	↓	0.75	0.80	0.71	0.17	0.24	0.21
Cholesterol	HMDB0000067	Cholesterols and derivatives	386.3549	27.25	3057	145, 329, 368, 458	1TMS	6	**0.020**	0.059	**0.003**	↓	0.77	0.77	0.77	0.34	0.26	0.30

AUC, area under the receiver operating characteristic curve (ROC); FC, fold change; HMD, human metabolome database; RT, retention time ^*^ *p*‐value corrected by Benjamini–Hochberg multiple postcorrection. Regulation: Variations observed for HBM of SARS‐CoV‐2 positive mothers against the control group: ↑ increase of the *m*/*z* signal, ↓ decrease of the *m*/*z* signal. The ^*^ *p*‐values corrected by Benjamini–Hochberg (*p* ˂ 0.05) are shown in the table in bold.

Nine metabolites (glycine, phenylalanine, tyrosine, proline, valine, isoleucine, uric acid, tocopherol, and cholesterol) were down‐regulated in SARS‐CoV‐2 mothers compared to controls while 11 were up‐regulated (inositol, 1,2,3‐propanetricarboxylic acid, pantothenic acid, glycerol, 9,12‐octadecadieno, phosphoric acid, meristic acid, 9‐hexadecenoic acid, 2‐monopalmitin, decanoic acid, and lauric acid) (Figure 2D). The over‐ or down‐regulation was also the same when comparing symptomatic and asymptomatic (Figures 2E,F, respectively) with the control group, but the fold change was not (Table 3).

### 2.4 Association between Human Milk Metals, Metabolites, and Ab against SARS‐CoV‐2

**Figure 3:**
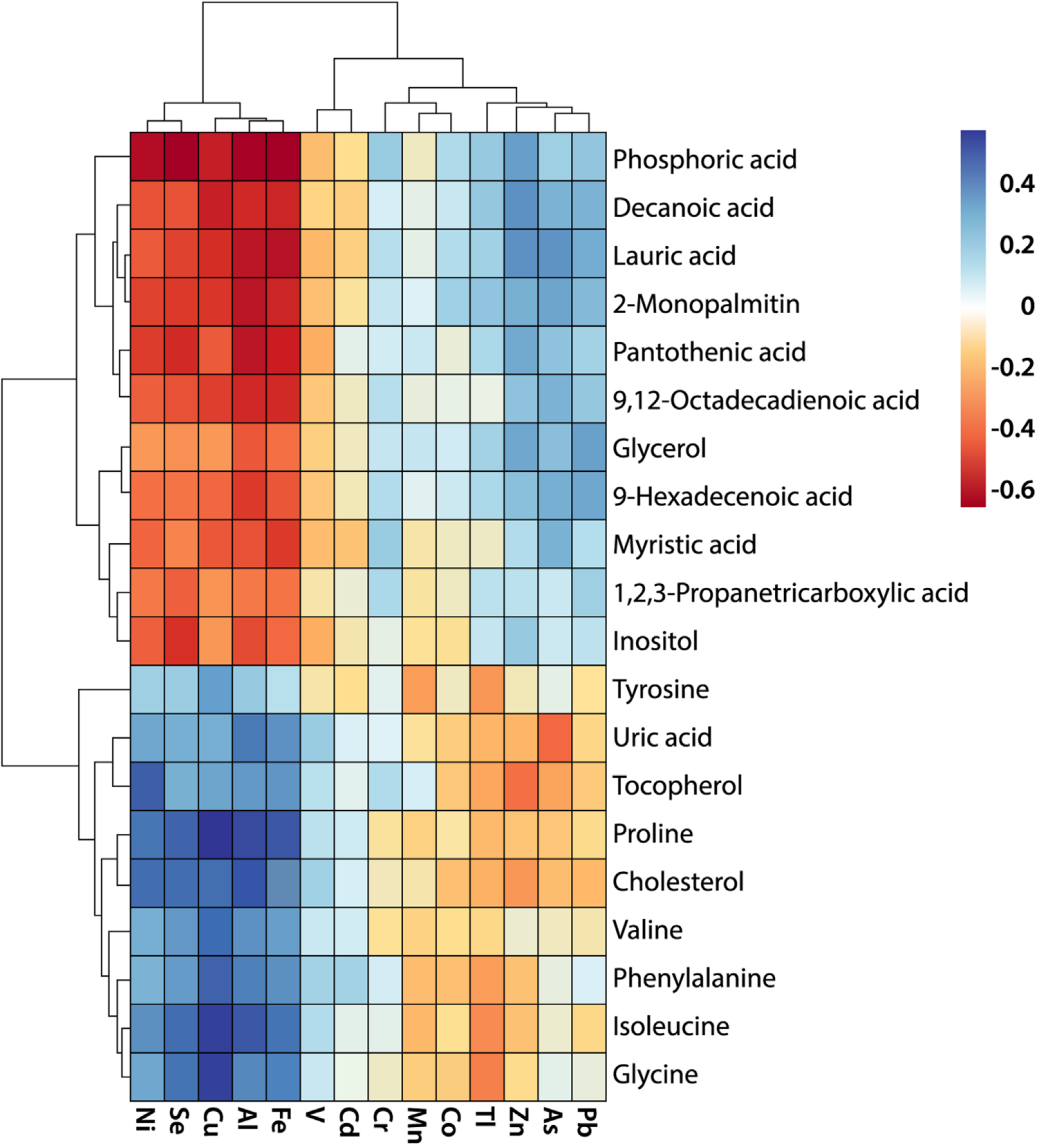
Spearman correlation heatmap analysis showing the link between elements and metabolites in HM from SARS‐CoV‐2 positive mothers.

We observed two groups of metals that present an opposite behavior against the same group of metabolites within SARS‐CoV‐2 women (**Figure** **3**). Likewise, the elements Cd, V, Ni, Se, Cu, Al, and Fe (group I) were positively correlated with tyrosine, uric acid, tocopherol, cholesterol, proline, valine, phenylalanine, glycine, and isoleucine, while the elements Pb, Arsenic (As), Zn, Tl, Cr, Co, and Mn (group II) were negatively correlated with the same metabolites. In addition, the elements of group I were negatively correlated with 2‐monopalmitin, lauric acid, decanoic acid, pantothenic acid octadecadienoic acid, phosphoric acid, inositol, propanetricarboxylic acid, myristic acid, 9‐hexadecenoic acid, and glycerol, while the group II of elements were positively correlated with the same metabolites. Table S4, Supporting Information collects the correlations that were significant (*p* < 0.005).

Furthermore, specific associations between the levels of SARS‐CoV‐2 immunoglobulins (Igs) such as IgA, IgG, and IgM and metals were identified (Figure S4, Supporting Information). Higher levels of IgA were associated with lower Se and Co (Rho = −0.57, *p* = 0.025 for both). Higher IgG levels were also associated with lower Se in milk (Rho = −0.54, *p* = 0.038) and also, lower Ni (Rho = −0.62, *p* = 0.014). Interestingly, no significant correlations between metals and Ig in asymptomatic COVID‐19 were identified, however higher IgA levels were not significantly associated with lower Zn (Rho = −0.46, *p* = 0.052), higher Cu was associated to higher IgG (Rho = 0.41, *p* = 0.09), and IgM (Rho = 0.42, *p* = 0.084). There were not significant associations between immunoglobulins and metabolites in the analyzed samples.

## 3 Discussion

COVID‐19 alters the elemental and metabolomic profile in HM samples with potential relevance for developing infant. Our results are, in our knowledge, among the first ones to describe the levels of elements and the metabolomic profile in HM samples from SARS‐CoV‐2 patients and their comparison with prepandemic controls. Furthermore, we analyzed the impact of COVID‐19 symptomatology as well as the impact at different lactational stages.

There are not data of elements in HM in the COVID‐19 context previously described in the literature. Most of the elements (Se, Ni, Fe, Cu, V, and Al) have significant lower concentrations in HM from COVID‐19 mothers compared to controls, while Zn and other minority elements as Tl and As were higher. We identified variability in the elemental profile of the women included in this study and also, the lactational stage was relevant too. There is not association between the levels in serum and HM for the main elements including Fe, Cu, and Zn, except for Se levels.^[^ ^17^ ^]^ It has been proposed that elements levels in milk are regulated by local secretion mechanisms.^[^ ^18^ ^]^ Previous studies in serum demonstrated lower concentrations of Ca, Se, Fe, and Zn in COVID‐19 patients, except Cu.^[^ ^8^ ^]^ Cu serum levels have been found moderately elevated in COVID‐19 survivors and this fact could be related with the response to infection^[^ ^19^ ^]^; as Cu is known as an acute phase reactant that increase with the inflammation.^[^ ^20^ ^]^ On the other side, Zn has been shown to possess antiviral activity through inhibition of SARS‐CoV‐2 replication in vitro^[^ ^21^ ^]^ and also, lower serum Zn levels have been reported in severe COVID‐19 patients compared to mild symptomatic ones.^[^ ^22^ ^]^ Furthermore, Zn deficiency has also been related with COVID‐19 complications that may be related with the modulatory effect of Zn on SARS‐CoV‐2 spike protein interaction with angiotensin converting enzyme 2 (ACE2).^[^ ^23^ ^]^ To date, although Zn supplementation has been pointed out to be efficient in COVID‐19 management,^[^ ^23^ ^]^ further trials are claimed. We found higher levels of Zn in HM from naturally infected women that could be potentially beneficial for the neonate. Fe levels in serum correlated with COVID‐19 severity^[^ ^24^ ^]^ and associated with inflammatory cytokine levels,^[^ ^25^ ^]^ while we found lower Fe levels in HM from COVID‐19 women. HM Se levels were also lower in COVID‐19 patients in agreement with previous data showing lower Se levels in serum samples of COVID‐19 patients compared to controls and inversely correlated with lung damage.^[^ ^8^ ^]^ Low serum levels of both, total serum Se and selenoprotein P have been associated with COVID‐19 mortality^[^ ^26^ ^]^ and Se supplementation is recommended, especially in deficient populations.^[^ ^27^ ^]^ Se is an antioxidant element that is involved in the immune function, cancer chemoprevention,^[^ ^28^ ^]^ and the metabolism of thyroid hormones.^[^ ^29^ ^]^ An excess or deficiency of trace essential elements can led to important health problems and for this reason, the homeostatic state should be carefully maintained within the body with a perfect balance of their concentrations in blood and organs,^[^ ^30^ ^]^ however, the potential impact for the lactating neonate is unknown.

In view of significant changes of HM elemental composition, the ratios between elements were also evaluated in the studied cohort. Only the Cu to Zn ratio (*p* = 0.001) and the Se to Zn ratio (*p* = 0.000) were significantly reduced in HM of COVID‐19 mothers compared to controls. This observation is different to serum data where the Cu to Zn ratio has been reported to increase gradually in association with COVID‐19 severity^[^ ^8^ ^]^ and it is considered the most successfully used predictor to distinguish between pathologies such as lung cancer and healthy controls.^[^ ^31^ ^]^ It has been demonstrated that the combined use of serum selenoprotein P and Zn along with age might be considered a reliable predictor of COVID‐19 survival.^[^ ^32^ ^]^ This fact corroborates the key role of Se and selenoprotein P in the modulation of redox homeostasis and endoplasmic reticulum stress, with subsequent regulation of inflammatory and immune signaling,^[^ ^33^ ^]^ as well as viral replication.^[^ ^34^ ^]^

It is also needed to highlight that the elemental composition of HM strongly depends on the lactation stage.^[^ ^12^ ^]^ Our study reported that COVID‐19 influenced the elemental profile at different lactational stages (Figure S3, Supporting Information), indicating that COVID‐19 affects the elemental distribution in any lactation stage with possible effects to the infant along lactation. However, no differences were observed depending on COVID‐19 symptomatology (asymptomatic vs symptomatic). Furthermore, we also identified toxic elements (Ni, V, Al, Tl, and As) in HM samples as reported by other studies^[^ ^15^, ^35^ ^]^ and, also, we reported their higher presence for first time in COVID‐19 mothers. Higher levels of Tl (1.4‐fold, *p* = 0.008) and As (1.3‐fold, *p* = 0.031) were identified in COVID‐19 mothers compared to prepandemic controls. Tl levels have been detected in all the samples at very low concentrations, this toxic has been also found naturally in the environment, and therefore can contaminate water and food as well as it was reported in Spanish HM in higher concentrations than our study.^[^ ^15^ ^]^ In agreement with other studies, the presence of As has been associated to residence in an urban area (higher availability of As in food, water, and air caused by industrial activities). The change of toxic elements concentrations in HM associated with COVID‐19, maybe explained by the homeostatic equilibria with other essential elements that could interact with them, as previously reported in the literature.^[^ ^36^ ^]^

In addition, we hypothesized that the Ab antibodies response against SARS‐CoV‐2 infection would be associated with specific elements. Higher anti‐SARS‐CoV‐2 levels of IgA were associated with lower Se and Co and higher IgG levels were also associated with lower Se and Ni in symptomatic women. Interestingly, Cu levels were associated (*p* > 0.05) with higher levels of IgG and IgM in milk. Taken all these differences and associations together, we can suggest different explanations. On the one hand, the SARS‐CoV‐2 infection itself as well as the physiological and immunological mechanisms initiated to control it^[^ ^37^ ^]^ may be responsible for such changes, involving their metabolic utilization, not only at systemic level, but also being reflected in their HM content, as happens in other infections or maternal disorders.^[^ ^38^, ^39^, ^40^, ^41^, ^42^, ^43^ ^]^ On the other hand, it would be plausible that those mothers with lower content of particular elements with key importance in the immune antiviral response such as Se, Ni, Fe, Cu, have been those more susceptible to infection and therefore displaying a different elemental pattern. This hypothesis is reinforced with the inverse association between the levels of certain elements and anti‐SARS‐CoV‐2 Ab, thus, the lower basal levels, the higher the infection and the corresponding humoral response. However, this does not explain the lack of differences related to symptomatology, and also the opposite effect of zinc in these samples.

Our study reported significant impact of SARS‐CoV‐2 infection on the metabolomic profile as well as the COVID‐19 symptomatology. We identified nine milk metabolites that were down‐regulated and 11 up‐regulation in HM from SARS‐CoV‐2 positive mothers compared to controls, being the aminoacyl‐tRNA biosynthesis, aromatic amino acid, and tryptophan metabolisms pathways the ones altered by the disease. Among them, stood out a modified metabolic profile associated to an alteration with down‐regulated in amino acids, cholesterol, and tocopherol while lineolic acids, fatty acids, and alcohols were associated to up‐regulated. It is noteworthy that the same metabolites are altered in HM from SARS‐CoV‐2 positive mothers with and without symptoms compared to prepandemic milk samples. To date, only one study investigates the metabolome of HM (colostrum) from SARS‐CoV‐2 positive mothers using liquid chromatography coupled to mass spectrometry.^[^ ^44^ ^]^ The study identified 504 lipids, and among them, just 13 lipids had significant changes between COVID‐19 patients and healthy controls. They also found 340 altered metabolites in HM from COVID‐19 mothers and also, they identified alterations in the aminoacyl‐tRNA biosynthesis, aromatic amino acid, and tryptophan metabolisms, in agreement with our data. The reason of the aminoacyl‐tRNA biosynthesis alteration is the amino acid depletion associated with COVID‐19, as described previously.^[^ ^45^ ^]^ In agreement with our data in milk, a recent study has demonstrated^[^ ^46^ ^]^ an alteration on the amino acid metabolism in serum samples from COVID‐19 patients.

*Tryptophan catabolism* and the metabolism of *tyrosine and phenylalanine* were also reduced in HM of COVID‐19 mothers, showing a *phenylethylamine* down‐regulation, which participates in the host‐microbe interplay by the immune system.^[^ ^44^ ^]^ Besides amino acids, *uric acid* (purine metabolism) levels also decreased in HM from COVID‐19 mothers. An inadequate metabolism of urea could be related with the sudden infant death syndrome (SIDS).^[^ ^47^ ^]^ A reduction in *tocopherol* levels was also found in COVID‐19 mothers and lower tocopherol concentration in HM has been associated with neonatal bronchopulmonary dysplasia, hemolytic anemia, neurological disorders, and neonatal mortality.^[^ ^48^ ^]^ Higher levels of *inositol* and *pantothenic* acid have been reported in COVID‐19 mothers. The increase of *glycerol* levels in HM of COVID‐19 mothers may be related with the breakdown of mono‐, di‐, and tri‐glycerides or glycerophospholipids, that are in the dendrites, myelin sheath, and synapses neural structures that are vital for brain connections.^[^ ^49^ ^]^ *Palmitoleic acid* was also elevated in HM from COVID‐19 mothers. This metabolite acts as a biochemical marker of fatty acid metabolism and demonstrated that a diet rich in palmitoleic acid negatively affects cholesterol homeostasis leading to increased LDL‐cholesterol and decreased HDL‐cholesterol.^[^ ^50^ ^]^ The metabolite *2‐monopalmitin* increased in HM of COVID‐19 mothers. Palmitic acid (16:0), which is absorbed as sn‐2 monopalmitin from HM, is the major saturated fatty acid in HM that accounts for 20–25% of HM fatty acids with 70% of the 16:0 esterified to the sn‐2 position of the milk triacylglycerol.^[^ ^51^ ^]^ Finally, *lauric acid* (fatty acid) was up‐regulated in HM of COVID‐19 mothers. In agreement with our data in COVID‐19 women, lower levels of capric and lauric acids have been reported in HM from mothers with cold‐like symptoms.^[^ ^52^ ^]^ It is well known that fat is one of the most important HM compounds because it is required for energy to infant growth and development assisting the metabolic and physiological functions of the infant body and that fatty acids are the hormones building blocks.^[^ ^53^ ^]^ We also reported specific associations between elements and metabolites suggesting a critical balance of those components in HM and the relevance of virus infection.

The results of this study revealed that HM from SARS‐CoV‐2 positive mothers present an elemental composition and a metabolomic profiles significantly altered than those observed in healthy controls. The metabolomic profile was also found to be different between the HM from symptomatic and asymptomatic COVID‐19 mothers. Despite the lower number of samples, we identified differences between active infection (PCR+) and seropositive women (past infection) as well as confirming the long impact effect during lactation of the virus infection. On the other side, it has been described that maternal diet would modify the mineral and metabolomic profile and dietary data was not collected in this study. Future studies considering maternal diet as well as other potential maternal‐neonatal factors warrant further studies. In addition, blood samples were not collected and compared with the HM results in order to identify the impact of COVID‐19 on elements and metabolites at systemic levels. Finally, metabolomic profiles could be affected by time as samples were collected with time difference between prepandemic and pandemic years.

Despite all those limitations, this study provides important insights about the impact of maternal SARS‐CoV‐2 infection on the elemental composition and metabolomic profile of HM that permits further research due the potential implications for maternal–infant health. Furthermore, the potential effect of COVID‐19 vaccines on HM composition needs to be ascertained and further studies are urgently needed.

## 4 Experimental Section

### Study Participants and Study Design

This was an observational and longitudinal study in Spanish mother–infant pairs with confirmed SARS‐CoV‐2 infection. The recruitment period was from April to December 2020 (pandemic samples) and from March to July 2015 (prepandemic samples). Participants were pregnant women intending to breastfeed, and nursing women with positive PCR for SARS‐CoV‐2 on nasopharyngeal swabs or presence of SARS‐CoV‐2 Ab in serum determined at the hospitals. Exclusion criteria included women unable to breastfeed due to severe symptomatology that required intensive care unit and/or mother's need for drugs with potential adverse effects on the infant and/or impossibility to obtain milk. A control group of women from prepandemic time and nonexposed to SARS‐CoV‐2 were also included. All participants received oral and written information about the study and written consent was obtained. All protocols performed in the study were in accordance with the ethical standards approved by the Ethical Committee of the Hospital Clínico Universitario of Valencia (ref. 2020/133), by CSIC Research (ref. 061/2021) and, by the Regional Ministry of Health and Families of Andalusia (ref. PI053/1), Spain. Clinical trial registration: https://www.clinicaltrials.gov/ct2/show/NTC04768244; Unique identifier: registered as NCT04768244.

### Human Milk Samples: Collection and Processing

A total number of 54 HM samples were included from both, healthy prepandemic control (*n* = 20) and COVID‐19 mothers (*n* = 34, *n* = 18 symptomatic, and *n* = 16 asymptomatic). HM samples were collected at different lactational stages: colostrum <7 days; transitional milk 7–14 days, and mature milk >14 days (Figure S1‐flow chart, Supporting Information).

HM collection was performed following a standardized protocol following the recommended procedures.^[^ ^54^ ^]^ In brief, breast skin was cleaned with water and soap and the first drops were discarded. Subsequently, milk was collected by using of a sterile pumper in sterile bottles to normalize the collection among participants. Morning collection was recommendable. Finally, HM samples were stored immediately at −20 °C in deep freezers and sent to the hospital to be stored at −80 °C until further analysis. Control milk samples were stored at −80 °C before processing.

### Elemental Analysis of Human Milk by ICP‐MS

A model MARS microwave oven (CEM Matthrews, NC, USA) and MiniXpress Polytetrafluoroethylene (PTFE) vessels were used for the mineralization of HM samples. To this end, an aliquot of 400 µL of each HM sample was placed into microwave PTFE vessels and weighted. Then, 4 mL of nitric acid and 1 mL hydrogen peroxide (4:1, v/v) were added. The PTFE vessels were closed after 10 min of premineralization and introduced in the microwave oven. The power was set at 400 W and a temperature program was applied from room temperature to 160 °C in 15 min and held at this temperature for 20 min. Finally, the elements were determined in HM samples by ICP‐MS (Agilent 8800 Triple Quad ICP‐MS; Agilent Technologies, Tokyo, Japan) using Rh at 100 ng L^–1^ as internal standard.

Isotopes monitored in the ICP‐MS analysis were ^27^Al, ^51^V, ^53^Cr, ^55^Mn, ^57^Fe, ^59^Co, ^60^Ni, ^63^Cu, ^65^Cu, ^64^Zn, ^66^Zn, ^75^As, ^78^Se, ^80^Se,^103^Rh, ^112^Cd, ^114^Cd, ^205^Tl, and ^208^Pb with dwell time of 0.3 s per isotope. A tuning aqueous solution of Li, Co, Y, and Tl at 1 µg L^–1^ was used to tune the ICP‐MS. A multielement calibration standard‐2A solution at 10 mg L^–1^ of Al, V, Cr, Mn, Fe, Co, Ni, Cu, Zn, As, Se, Cd, Tl, and Pb was obtained from Agilent Technologies. Most of the analyzed elements required 4.5 mL min^–1^ flow‐rate of helium. For Se, a mixture of H_2_ (2 mL min^–1^) and O_2_ (40%) was used in the MS/MS mode.

The experiments were performed in triplicates (three different HM samples were taken). The quality control validation parameters investigated were the limits of detection (LODs), limits of quantification (LOQs), precision, and accuracy (Table S1, Supporting Information). The LOD for each element was calculated by three times the standard deviation of the procedural blank from microwave digestion (*n* = 10). LOQ was determined by 10 times the standard deviation of the procedural blank. Element values below LOD were excluded from further statistical evaluations. The precision was determined in terms of the percent of relative standard deviation (% RSD). The accuracy (% recovery) was determined by the recovery experiments for the 14 selected elements which were spiked at two different concentration levels of 10 and 100 ng g^–1^. To verify the accuracy of the ICP‐MS method, a milk powder certified reference material (CRM) (NIST‐1849) was also analyzed for the determination of the elements such as ^80^Se, ^78^Se, ^66^Zn, ^64^Zn, ^65^Cu, ^63^Cu, ^57^Fe, ^55^Mn, and ^53^Cr. Other operational parameters were collected in Table S2, Supporting Information.

### Untargeted Metabolomic Profile of Human Milk by GC‐MS

For the metabolomic analysis, a model Trace GC ULTRA gas chromatograph coupled to a model ITQ900 mass spectrometer (Thermo Fisher Scientific, Bremen, Germany) was used. The chromatographic column model VF‐5MS Factor Four (30 m × 0.25 mm ID, 0.25 µm of film thickness, Agilent Technologies) was mounted into the GC. 50 µL whole milk was vortex‐mixed with 175 µL of methanol and 175 µL of methyl tert‐butyl ether (MTBE) and then, centrifuged at 4000 × *g* during 15 min at 15 °C. The sample extraction protocol was previously published by Villaseñor et al.^[^ ^55^ ^]^ Briefly, an aliquot of 150 µL was transferred into a glass vial, dried in a speed vacuum system (SpeedVacTM Thermo Scientific, Waltham, MA, USA) during 1 h at 45 °C, and reconstituted with the derivatizing agents. For protection of carbonyl groups by methoxymation, dried extracts were redissolved in 50 µL of 20 mg mL^−1^ methoxyamine in pyridine and after briefly vortexing, they were incubated at 80 °C for 15 min in a water bath. Subsequently, silylation was performed by adding 50 µL of MSTFA followed by incubation at 80 °C for 15 min. Finally, extracts were centrifuged at 2039 × *g* for 5 min and the supernatants collected for analysis. A total of 10 quality control samples (QCs) were prepared by pooling equal volumes of all samples studied, which were injected in the beginning and at the end of each batch sequence to check systems stability and performance of the analysis.

Chromatographic separation of metabolites was carried out by a temperature program in the GC‐MS oven. An initial temperature was set to 60 °C, maintained during 1 min and increased to 325 °C, at 10 °C min^–1^. The injector temperature was set at 280 °C and a constant carrier gas flow‐rate of 1 mL min^−1^ of He was used. The total chromatographic time required for the separation was 30 min. Ionization of metabolites was performed at 70 eV by electronic impact (EI) and they were detected in the ion trap that operated in full scan mode screening the range 35−650 *m*/*z*, with 230 °C as ion source temperature.

### Human Milk Antibodies against SARS‐CoV‐2

HM specific IgA, IgM, and IgG antibody levels targeted to the receptor‐binding domain (RBD) of the SARS‐CoV‐2 spike protein were included from the previous COVID‐19 study.^[^ ^3^ ^]^ Briefly, the analysis was carried out as follows: 96‐well immunoplates (Corning, Kennebunk, ME, USA) were coated with 2 µg mL^−1^ of RBD overnight at 4 °C, blocked in 3 % (w/v) milk powder in PBS containing 0.1% Tween 20. Samples were incubated at a 1:4 dilution, incubated for 2 h and after three washing steps incubated with antihuman IgA (α‐chain‐specific) horseradish peroxidase (HRP) antibody (Thermo‐Fisher Scientific, Bremen, Germany; # A18781; 1:6000), antihuman IgM (µ‐chain‐specific) HRP antibody (Sigma‐Aldrich, St. Louis, MO, USA; # A0420; 1:4000), and antihuman IgG (Fc‐specific) HRP antibody (Sigma‐Aldrich, St. Louis, MO, USA; # A0170; 1:4000) for 1 h. Plates were developed with 3,3′,5,5′‐Tetramethylbenzidine and reactions were stopped with 50 µL of 2 M sulfuric acid. Absorbance at 450 nm was read in a ClarioStar (BMG Labtech, Ortenberg, Germany) microplate reader.

### Statistical Analysis

All data were analyzed using Statistica 8 (Statsoft, Tulsa, OK, USA). The obtained data were expressed as mean and the respective standard error of the mean (S.E.M.) of the elements (Mean ± S.E.M.). Data related with element concentrations of HM, were analyzed by nonparametric methods since most of the variables showed a skewed distribution (checked by normal probability plots) and variances were not homogeneous (checked by Levene´s test). Group comparisons were performed using Mann–Whitney *U* test. Only *p* values below 0.05 were regarded as statistically significant. Finally, the performance characteristics of the elements were evaluated and compared in terms of the area under the receiver operating characteristic curve (AUC values from ROC). In addition, Orthogonal Partial Least Squares–Discriminant Analysis (OPLS‐DA) and heatmaps were performed to delve the potential contribution of the elemental composition and metabolites present in HM to discriminate the studied groups. ROC curves, OPLS‐DA and heatmaps were obtained using MetaboAnalyst version 5.0 (https://www.metaboanalyst.ca/).

Data processing in GC–MS was performed with the free accessible R platform software (http://www.rproject.org). The GC–MS data matrix, including t_R_‐m/z pair, sample names, and normalized peak area, was imported into the SIMCA‐P (version 11.5, Umetrics). Principal component analysis (PCA) and orthogonal partial least square discriminant analysis (OPLS‐DA) were used to establish predictive models to visually display metabolic information. Candidate metabolites (VIP ≥1.5) were found by the OPLS‐DA model. In addition, the fold change was calculated for each metabolite estimate the variation in the abundance of the metabolites within each comparison. The values of class separation (*R* ^2^) and predictive power (*Q* ^2^) were used to assess the quality of the model. Features were filtered by selecting those that were present in 80% for GC‐MS. Samples under study were classified by OPLS‐DA model using the intensities obtained of the *m*/*z* signals of all the mass spectra. Outliers and abnormal values were not observed in the PCA score plots and the QCs were grouped indicating low variability and good reproducibility of the measurement.

The differences between groups of samples were evaluated for each individual metabolite by One‐way ANOVA for any of the comparisons, and differences among the means were compared using Tukey's test in order to find which comparisons were statistically significant and to investigate the trend of the metabolite concentration. For multiple comparison corrections, the Benjamini–Hochberg (FDR correction) method was applied to all *p* values to control the false positive rate at level *α* >0.05. The National Institute of Standards and Technology (NIST) database was used for metabolite identification. Those with spectrum score >80% and concordant retention index (n‐alkane scale) were putatively annotated according to NIST. In addition, the area qualifier/target ion ratio per metabolite was checked to choose those with a variation less than 20%. Before any statistical calculation, sample concentrations were normalized by internal standard (IS) abundance to minimize the response variability introduced by the instrument. Moreover, data were filtered by coefficient of signal variation (CV) in quality controls (QCs), considering values lower than 30% as acceptable. Only the signals with VIP >1.5, fold changes between −2 and +2 and *p‐*values ≤0.05 were considered. Metabolites with AUC values ≥0.75 were selected as the most important HM biomarkers for SARS‐CoV‐2 positive mothers. Finally, metabolic pathways were analyzed by MetaboAnalyst 4.0 (http://www.metaboanalyst.ca/) online data system. Pathway enrichment analysis was performed using the “Homo sapiens” library with the “Hypergeometric Test” and the pathway topological analysis with the “Relative‐Betweenness Centrality” algorithms (impact value threshold = 0.1). HM Ig‐antibodies against SARS‐CoV‐2 levels were used to combine with metal and metabolites levels in order to identify potential associations between antibodies and specific elements and metabolites. Spearman rank correlation was used and *p* < 0.05 was considered statistically significant.

## Conflict of Interest

The authors declare no conflict of interest.

## Author Contributions

A.A.‐B., F.J.S.C., M.S.‐R. contributed equally to this work. M.C.C. and T.G.‐B. are senior authors and equal contributors. The authors’ responsibilities were as follows—T.G.‐B., M.C.C., and C.M.‐C.: designed the research (project conception and development of the overall research plan); C.M.‐C., E.G.V., C.L., I.V.L.: responsible of clinical study, recruitment, and collection of the biological samples; A.A.‐B., F.J.S.C., M.S.‐R., and C.B. analyzed milk samples; F.P.‐C., C.L.: supervised the study and assisted in the interpretation of the data; T.G.‐B. and C.M.‐C.: drafted the first draft; and all authors have read and approved the final manuscript.

## Supporting information

Supporting Information

## Data Availability

The data that supports the findings of this study are available in the supplementary material of this article.
