# Supplementary material for: Metallomic and Untargeted Metabolomic Signatures of Human Milk from SARS‐CoV‐2 Positive Mothers
Source: Mol Nutr Food Res. 2022 Jun 29;66(16):2200071. doi: 10.1002/mnfr.202200071 (PMC9350005; doi:10.1002/mnfr.202200071)
Supplement: Supplementary file 1 — Supporting Information [file MNFR-66-0-s001.pdf]

SUPPLEMENTARY DATA

Table S1. Averaged concentrations (ng g^{-1}) and quality control validation parameters obtained by ICP-MS: limits of detection (LODs), limits of quantification (LOQs), p-value* between group and recovery (%).

**Averaged Concentration (ng g^{-1}) ± S.E.M**	**Al**	**V**	**Cr**	**Mn**	**Fe**	**Co**	**Ni**	**Cu**	**Zn**	**As**	**Se**	**Tl**	**Pb**	**Cd**
**Group sample= CV-s (N=18)**	15.19±0.76	0.44±0.34	8.65±0.35	7.71±0.33	66.85±0.38	0.43±0.67	16.78±0.36	57.67±0.49	3482.54±0.54	1.3±0.36	7.47±0.16	0.26±0.42	4.04±0.18	0.48± 0.64
**Group sample= CV-a (N=16)**	46.23±0.25	0.67±0.22	8.42±0.27	8.85±0.29	100.86±0.21	0.34±0.86	29.38±0.22	75.50±0.29	4441.03±0.40	1.74±0.26	8.56±0.11	0.28±0.40	4.42±0.16	0.51±0.61
**Group sample=C (N=20)**	348.67±0.04	0.72±0.20	7.65±0.30	8.24±0.30	883.58±0.04	0.31±0.92	144.82±0.23	741.39±0.03	2324.56±0.78	1.17±0.36	17.42±0.06	0.19±0.56	4.05±0.56	0.45±0.31
**Upper Interval for Mean**														
**Group sample= CV-s**	45.31	0.76	12.94	12.07	126.35	1.27	29.80	137.84	6723.51	2.09	9.48	0.43	4.87	4.87
**Group sample= CV-a**	163.27	1.16	10.63	12.16	298.01	0.46	164.28	228.42	5929.23	2.99	9.80	0.43	9.06	9.06
**Group sample=C**	548.08	1.41	11.96	15.85	2419.88	0.89	732.54	3809.26	3677.16	3.08	25.47	0.26	7.15	7.15
**Lower Interval for Mean**														
**Group sample= CV-s**	0.00	0.24	0.01	3.90	31.23	0.20	9.66	14.89	771.49	0.53	4.38	0.12	2.44	2.44
**Group sample= CV-a**	7.54	0.23	5.21	4.35	43.08	0.15	5.73	23.96	1098.05	0.94	7.42	0.12	2.35	2.35
**Group sample=C**	115.00	0.39	4.03	4.82	361.17	0.00	6.19	192.33	1075.60	0.16	8.92	0.05	1.81	1.81
**p-value**														
**CV-s *vs* C**	0.000	0.001	0.38	0.74	0.000	0.299	0.002	0.000	0.045	0.807	0.000	0.107	0.995	0.915
**CV-a *vs* C**	0.000	0.872	0.78	0.88	0.000	0.968	0.006	0.000	0.000	0.009	0.000	0.012	0.567	0.694
**CV-a vs CV-s**	0.709	0.005	0.83	0.49	0.939	0.484	0.961	0.978	0.079	0.047	0.467	0.588	0.525	0.905
**LOD (ng g^{-1})**	0.09	0.02	0.20	0.05	0.02	0.01	0.04	0.02	0.64	0.006	0.03	0.01	0.004	0.05
**LOQ (ng g^{-1})**	1.41	0.12	0.44	0.13	0.22	0.10	0.14	0.16	1.47	0.01	0.11	0.10	0.01	0.10
**Recovery (%)**	96.14	97.08	99.01	98.21	95.43	101.20	99.10	101.76	98.21	99.40	94.46	97.45	98.86	97.70

*p-values were calculated applying Tukey test. The significant p-values ($p<0.05$) are shown in the table in red colour.

Table S2. Instrumental conditions for ICP-QQQ-MS analysis

**ICP-MS/MS parameters**	
Forward Power	1600 W
Plasma gas flow	15 L min^{-1}
Auxiliary gas flow	1 L min^{-1}
Carrier gas flow	0.650L min^{-1}
Sampling depth	10 mm
Nebulizer type	MicroMist
H_2 gas flow	2.00 mL min^{-1} (40% O_2)
Torch	Shield (with long life platinum shield plate
OctP Bias	-18.0 V
OctP RF	190 V
Energy Discrimination	-7.0 V
Integration time	0.3 s
Replicates	5
Isotopes monitored	^{27}Al,^{51}V, ^{53}Cr, ^{55}Mn, ^{57}Fe, ^{57}Fe, ^{59}Co, ^{60} Ni, ^{63}Cu, ^{65} Cu,^{64}Zn, ^{66}Zn, ^{75}As , ^{78}Se, , ^{80}Se, , ^{103}Rh, ^{112}Cd, ^{114}Cd ,^{205} Tl, ^{208}Pb

Table S3. Averaged concentrations, Mean ± S.E.M (ng g^{-1}), of elements in HM from CV-s, CV-a and from healthy controls at different lactation stages.

Group	Sub-group	Lactation Stage	Al	V	Cr	Mn	Fe	Co	Ni	Cu	Zn	As	Se	Tl	Pb	Cd
		**Colostrum** N=6	16.58±0.98	0.39±0.39	8.8±0.26	7.75±0.39	66.76±0.37	0.27±0.2	17.41±0.35	54.06±0.35	4041.78±0.55	1.45±0.38	7.60±0.10	0.22±0.65	4.01±0.25	0.52±0.94
**CV**	cv-s	**Transitional milk** N=6	13.46±0.91	0.52±0.39	8.48±0.37	6.75±0.46	57.37±0.47	0.69±0.61	16.07±0.55	68.37±0.33	3304.54±0.51	1.0±0.57	7.39±0.18	0.33±0.31	4.08±0.12	0.47±0.39
		**Mature milk** N=6	15.79±0.34	0.44±0.17	9.11±0.19	6.98±0.26	64.72±0.25	0.37±0.39	15.97±0.33	54.17±0.39	2602.25±0.72	1.35±0.08	7.25±0.14	0.24±0.23	3.94±0.16	0.51±0.33
		**Colostrum** N=8	49.24±0.48	0.73±0.39	9.15±0.12	9.62±0.20	99.87±0.37	0.37±0.16	34.68±1.53	71.38±0.40	4846.08±0.21	1.68±0.25	8.67±0.08	0.34±0.35	3.92±0.24	0.60±0.66
**CV**	CV-a	**Transitional milk** N=4	48.01±1.60	0.58±0.41	6.92±0.36	7.2±0.34	114.3±1.07	0.28±0.36	18.63±0.42	79.83±1.06	4529.78±0.19	1.92±0.44	8.47±0.12	0.23±0.58	5.63±0.41	0.31±0.46
		**Mature milk** N=4	42.60±0.42	0.69±0.03	7.71±0.23	8.21±0.20	91.57±0.39	0.30±0.18	16.45±0.29	72.16±0.10	3599.28±0.60	1.93±0.06	8.46±0.06	0.25±0.32	4.55±0.28	0.46±0.33
		**Colostrum** N=7	324.45±0.47	0.70±0.27	7.64±0.29	9.42±0.53	968.2±0.73	0.29±0.95	146.86±0.94	462.56±0.37	1982.82±0.33	1.25±0.74	20.91±0.17	0.19±0.37	4.37±0.52	0.45±0.48
**C**	--	**Transitional milk** N=6	396.54±0.45	0.71±0.51	7.60±0.47	7.73±0.22	822.8±0.28	0.34±0.55	185.33±1.46	491.05±0.50	2722.78±0.22	1.18±0.67	16.70±0.17	0.19±0.21	3.89±0.29	0.45±0.24
		**Mature milk** N=7	331.87±0.37	0.75±0.18	7.71±0.29	7.51±0.27	851.1±0.45	0.31±0.43	108.06±0.86	806.23±0.97	2324.97±0.21	1.08±0.46	14.55±0.25	0.19±0.16	3.92±0.18	0.47±0.18

Table S4. Spearman correlation coefficients between element and metabolites significative.

Samples=C *vs* CV	Al		V		Cr		Mn		Fe		Co		Ni		Cu		Zn		As		Se		Cd		Tl		Pb	
Metabolites	R	p-value	R	p-value	R	p-value	R	p-value	R	p-value	R	p-value	R	p-value	R	p-value	R	p-value	R	p-value	R	p-value	R	p-value	R	p-value	R	p-value
Valine	0.351	0.010	0.063	0.653	-0.124	0.377	-0.166	0.235	0.314	0.022	-0.124	0.378	0.274	0.047	0.433	0.001	-0.045	0.748	-0.065	0.642	0.336	0.014	0.050	0.720	-0.148	0.290	-0.083	0.556
Glycerol	-0.453	0.001	-0.169	0.226	0.082	0.559	0.050	0.723	-0.396	0.003	0.051	0.719	-0.310	0.024	-0.307	0.025	0.296	0.031	0.230	0.098	-0.329	0.016	-0.078	0.579	0.165	0.237	0.312	0.023
Isoleucine	0.486	0.000	0.125	0.374	-0.057	0.688	-0.238	0.086	0.422	0.002	-0.121	0.387	0.356	0.009	0.533	0.000	-0.212	0.128	-0.047	0.736	0.439	0.001	-0.004	0.975	-0.337	0.014	-0.151	0.281
Proline	0.514	0.000	0.100	0.477	-0.114	0.418	-0.169	0.225	0.483	0.000	-0.094	0.504	0.416	0.002	0.561	0.000	-0.205	0.141	-0.197	0.157	0.459	0.001	0.056	0.688	-0.230	0.097	-0.142	0.312
Glycine	0.375	0.006	0.069	0.625	-0.058	0.682	-0.179	0.199	0.385	0.004	-0.172	0.217	0.298	0.030	0.535	0.000	-0.133	0.344	-0.010	0.946	0.423	0.002	-0.015	0.913	-0.362	0.008	-0.029	0.837
Decanoic acid	-0.560	0.000	-0.159	0.255	0.033	0.815	-0.012	0.930	-0.571	0.000	0.073	0.606	-0.459	0.001	-0.588	0.000	0.351	0.010	0.265	0.055	-0.499	0.000	-0.173	0.217	0.199	0.154	0.262	0.058
Phenylalanine	0.394	0.003	0.169	0.228	0.037	0.791	-0.228	0.100	0.355	0.009	-0.213	0.126	0.262	0.058	0.458	0.001	-0.213	0.125	-0.020	0.889	0.328	0.016	-0.067	0.634	-0.301	0.028	0.018	0.896
Lauric acid	-0.616	0.000	-0.235	0.090	0.121	0.389	-0.014	0.920	-0.623	0.000	0.128	0.361	-0.443	0.001	-0.546	0.000	0.351	0.010	0.344	0.012	-0.498	0.000	-0.181	0.194	0.172	0.218	0.280	0.042
Phosphoric acid	-0.668	0.000	-0.216	0.120	0.191	0.171	-0.062	0.661	-0.680	0.000	0.139	0.322	-0.637	0.000	-0.590	0.000	0.317	0.021	0.176	0.207	-0.679	0.000	-0.133	0.344	0.194	0.165	0.207	0.137
Propanetricarboxylic acid	-0.378	0.005	-0.089	0.526	0.140	0.318	-0.106	0.450	-0.389	0.004	-0.067	0.632	-0.378	0.005	-0.317	0.021	0.104	0.460	0.064	0.649	-0.432	0.001	-0.038	0.789	0.102	0.468	0.177	0.206
Tyrosine	0.193	0.167	-0.094	0.504	-0.007	0.963	-0.301	0.028	0.112	0.426	-0.061	0.663	0.177	0.204	0.315	0.022	-0.078	0.579	-0.013	0.927	0.186	0.183	-0.125	0.372	-0.313	0.023	-0.116	0.408
Pantothenic acid	-0.616	0.000	-0.257	0.063	0.042	0.763	0.061	0.664	-0.581	0.000	-0.038	0.785	-0.513	0.000	-0.445	0.001	0.292	0.034	0.217	0.119	-0.558	0.000	-0.010	0.943	0.144	0.304	0.166	0.235
Inositol	-0.480	0.000	-0.259	0.061	-0.021	0.880	-0.120	0.391	-0.413	0.002	-0.032	0.819	-0.429	0.001	-0.313	0.022	0.197	0.158	0.061	0.665	-0.540	0.000	-0.089	0.527	0.077	0.584	0.095	0.498
9-Hexadecenoic acid	-0.517	0.000	-0.193	0.167	0.129	0.356	0.002	0.990	-0.457	0.001	0.057	0.687	-0.396	0.003	-0.414	0.002	0.224	0.106	0.276	0.046	-0.390	0.004	-0.087	0.535	0.146	0.296	0.297	0.031
Uric acid	0.405	0.003	0.187	0.179	0.009	0.947	-0.116	0.407	0.351	0.010	-0.179	0.200	0.294	0.033	0.410	0.002	-0.249	0.072	-0.410	0.002	0.279	0.043	0.029	0.836	-0.248	0.074	-0.153	0.273
Octadecadienoic acid	-0.564	0.000	-0.193	0.167	0.119	0.394	-0.026	0.855	-0.556	0.000	-0.020	0.888	-0.438	0.001	-0.506	0.000	0.215	0.122	0.260	0.060	-0.465	0.000	-0.071	0.614	0.170	0.222	0.198	0.155
Myristic acid	-0.463	0.000	-0.223	0.109	0.190	0.172	-0.095	0.499	-0.519	0.000	-0.071	0.613	-0.419	0.002	-0.447	0.001	0.128	0.360	0.264	0.056	-0.354	0.009	-0.213	0.126	-0.056	0.692	0.125	0.373
2-Monopalmitin	-0.617	0.000	-0.215	0.122	0.079	0.575	0.017	0.901	-0.573	0.000	0.172	0.219	-0.499	0.000	-0.529	0.000	0.273	0.048	0.304	0.027	-0.518	0.000	-0.118	0.399	0.209	0.133	0.246	0.075
Tocopherol	0.334	0.015	0.105	0.455	0.131	0.348	0.036	0.796	0.342	0.012	-0.195	0.162	0.471	0.000	0.300	0.029	-0.399	0.003	-0.287	0.037	0.270	0.051	0.000	0.997	-0.280	0.042	-0.187	0.180
Cholesterol	0.476	0.000	0.163	0.245	-0.075	0.592	-0.091	0.519	0.389	0.004	-0.218	0.117	0.438	0.001	0.362	0.008	-0.316	0.021	-0.223	0.109	0.430	0.001	0.038	0.786	-0.257	0.063	-0.236	0.090

Significant correlation coefficients ($p<0.05$) are shown in the table in red colour.

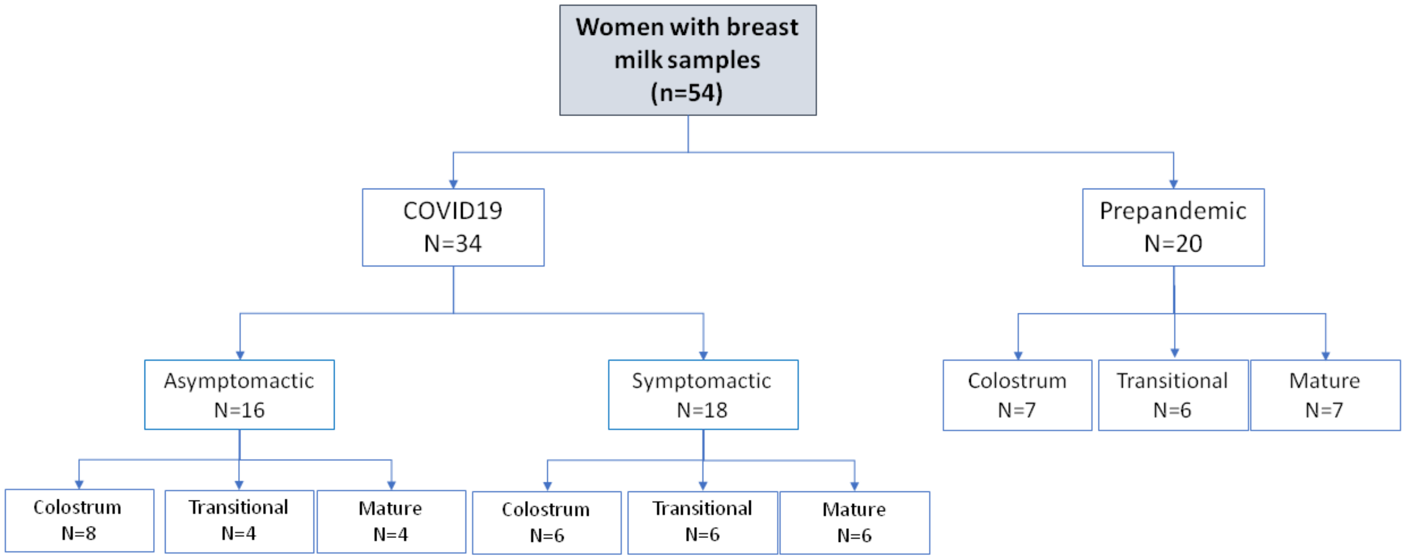

Figure S1. Flow chart of the human milk collection.

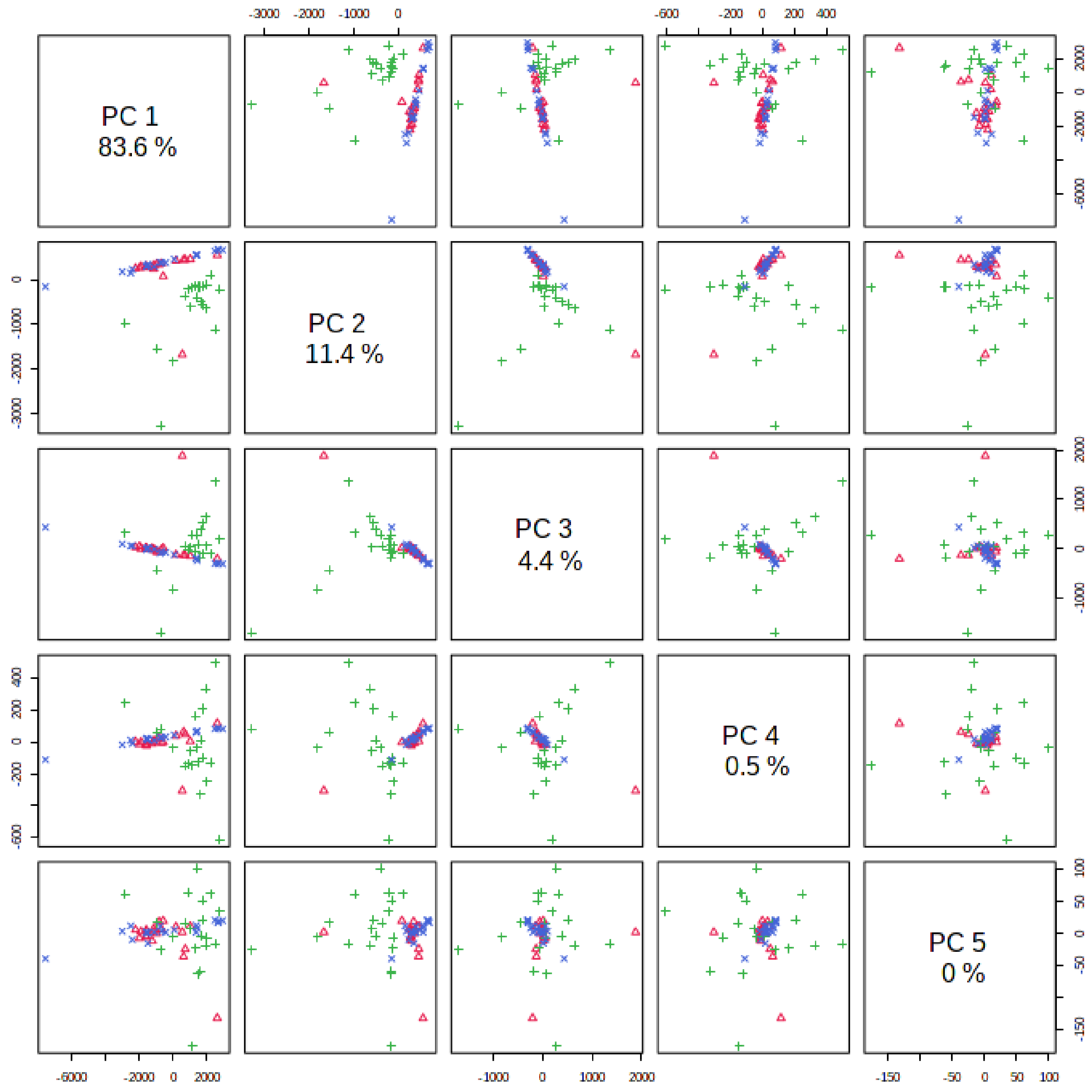

Figure S2. Pairwise score plots between the selected PCs based on the elemental composition of the HBM under study between COVID-19 mothers compared to healthy controls. The explained variance of each PC is shown in the corresponding diagonal cell.

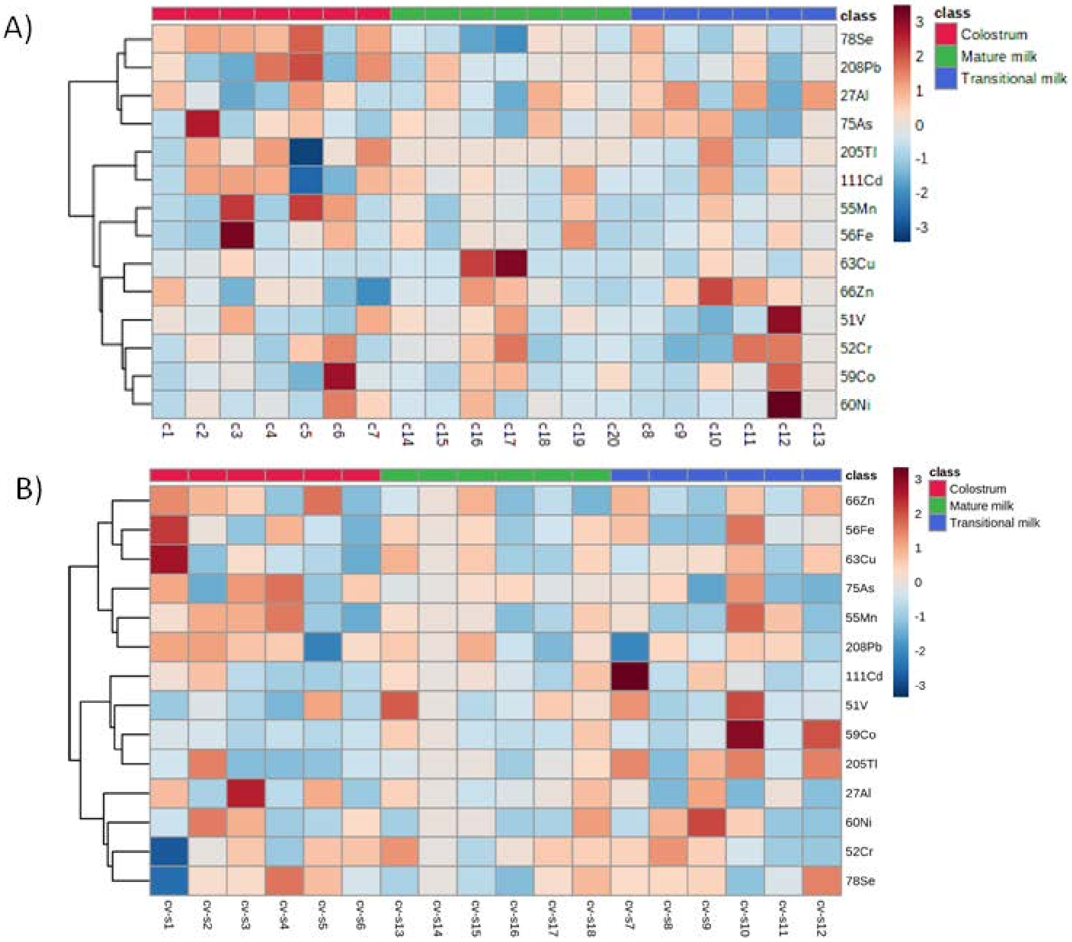

Figure S3. Heatmaps of HM samples from control (A) and symptomatic SARS-Cov-2 positive mothers (B) using the elemental composition along the different lactation stages.

A)

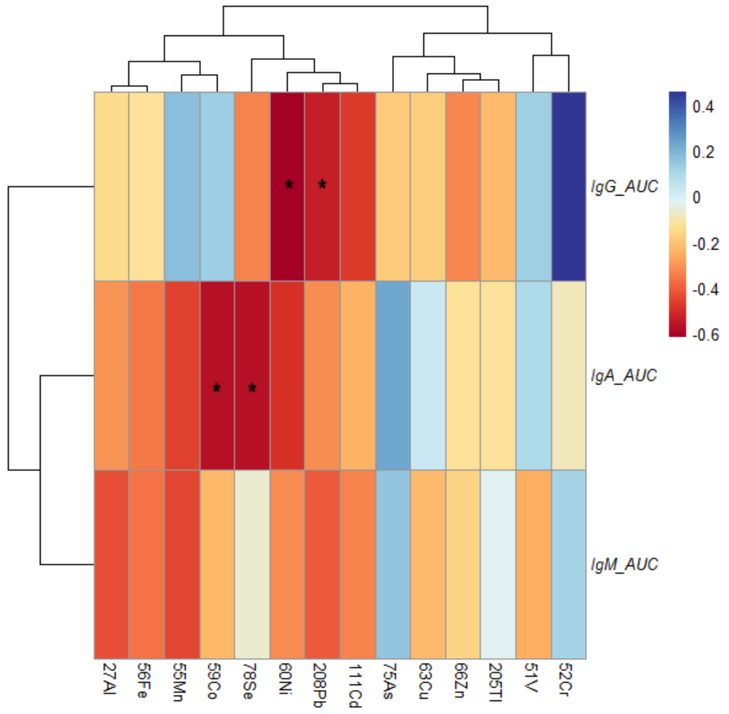

B)

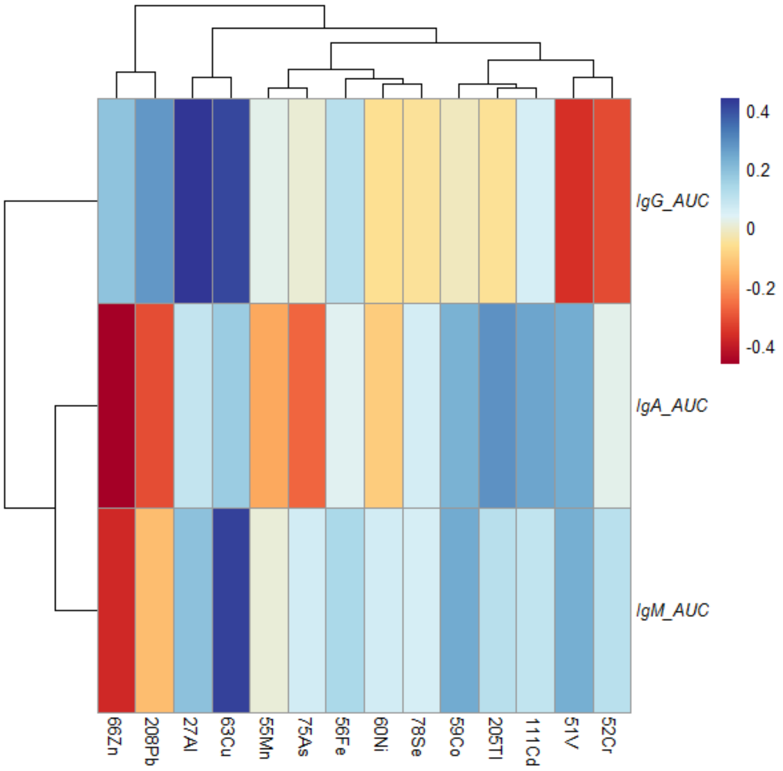

Figure S4. Heatmaps showing the correlation between elements and immunoglobulins. A) Symptomatic SARS-Cov-2 positive mothers, B) asymptomatic SARS-Cov-2 positive mothers. Association is significant when $p<0.005$.
